# The antagonistic strain *Bacillus subtilis* UMAF6639 also confers protection to melon plants against cucurbit powdery mildew by activation of jasmonate-and salicylic acid-dependent defence responses

**DOI:** 10.1111/1751-7915.12028

**Published:** 2013-01-10

**Authors:** Laura García-Gutiérrez, Houda Zeriouh, Diego Romero, Jaime Cubero, Antonio Vicente, Alejandro Pérez-García

**Affiliations:** 1 Instituto de Hortofruticultura Subtropical y Mediterránea ‘La Mayora’ (IHSM-UMA-CSIC), Departamento de Microbiología, Universidad de MálagaBulevar Louis Pasteur 31 (Campus Universitario de Teatinos), 29071, Málaga, Spain; 2 Instituto Nacional de Investigación y Tecnología Agraria y Alimentaria (INIA)Ctra de La Coruña km 7.5, 28040, Madrid, Spain

## Abstract

Biological control of plant diseases has gained acceptance in recent years. *Bacillus subtilis* UMAF6639 is an antagonistic strain specifically selected for the efficient control of the cucurbit powdery mildew fungus *Podosphaera fusca*, which is a major threat to cucurbits worldwide. The antagonistic activity relies on the production of the antifungal compounds iturin and fengycin. In a previous study, we found that UMAF6639 was able to induce systemic resistance (ISR) in melon and provide additional protection against powdery mildew. In the present work, we further investigated in detail this second mechanism of biocontrol by UMAF6639. First, we examined the signalling pathways elicited by UMAF6639 in melon plants, as well as the defence mechanisms activated in response to *P. fusca*. Second, we analysed the role of the lipopeptides produced by UMAF6639 as potential determinants for ISR activation. Our results demonstrated that UMAF6639 confers protection against cucurbit powdery mildew by activation of jasmonate- and salicylic acid-dependent defence responses, which include the production of reactive oxygen species and cell wall reinforcement. We also showed that surfactin lipopeptide is a major determinant for stimulation of the immune response. These results reinforce the biotechnological potential of UMAF6639 as a biological control agent.

## Introduction

Biological control, i.e. the use of natural enemies to combat pests or plant diseases, has gained acceptance in recent years. Among the different microbial species examined for that purpose, some aerobic spore-forming bacteria possess several advantages that make them good candidates for biological control agents. First, they produce several different types of insecticidal and antimicrobial compounds. Second, *Bacillus* species are able to produce spores that allow them to resist adverse environmental conditions and permit easy formulation and storage of commercial products (Schallmey *et al*., 2004; Francis *et al*., 2010). *Bacillus*-based biopesticides are widely used in conventional agriculture and represent the most important class of microbial products commercially available for phytosanitary use. In contrast, implementation of *Bacillus*-based biofungicides is still a pending issue (Pérez-García *et al*., 2011).

Powdery mildew diseases are one of the most important plant pathological problems worldwide. Important crops, including cereals, grapevine and a number of vegetables and ornamentals are among their major targets (Agrios, 2005). In crop protection, the largest area of fungicide use is for the control of powdery mildews (Hewitt, 1998). The impact of chemical control, however, has been tempered by the ease with which powdery mildew fungi (*Erysiphales*) have developed resistance to many systemic fungicides (Hollomon and Wheeler, 2002). The need for new control strategies for the management of powdery mildews has led researchers and growers to explore suitable environmentally friendly alternatives or complements to chemicals, biological control being the most investigated of these approaches (Bélanger and Labbé, 2002).

Powdery mildew fungi are ectoparasites, and therefore, they are perfect targets for antibiotic-producing bacteria. *Bacillus subtilis* UMAF6639 is an antagonistic strain specifically selected for its efficient control of the cucurbit powdery mildew fungus *Podosphaera fusca* (Romero *et al*., 2004), one of the most important limiting factors for cucurbit production worldwide (Pérez-García *et al*., 2009). The antagonistic activity of UMAF6639 mostly relies on the production of the antifungal lipopeptide iturins and fengycins (Romero *et al*., 2007b). These are amphiphilic compounds that exert their action by targeting fungal membranes, leading to the lysis of the fungal cells (Romero *et al*., 2007a). The field performance of UMAF6639 has also been tested, offering an excellent control for cucurbit powdery mildew on greenhouse-grown melon (Romero *et al*., 2007c). Recently, it was shown that iturins produced by UMAF6639 also disrupt bacterial membranes, thus providing additional control potential against the cucurbit pathogenic bacteria *Pectobacterium carotovorum* and *Xanthomonas campestris* (Zeriouh *et al*., 2011).

Biocontrol agents may face pathogens by means of multiple mechanisms. Some rhizosphere inhabitants such as mycorrhizae and plant-growth-promoting rhizobacteria (PGPR) can confer the plant an enhanced defensive capacity against a broad spectrum of fungal, bacterial and viral diseases by means of a phenomenon known as induced systemic resistance (ISR) (van Loon *et al*., 1998; Pozo and Azcón-Aguilar, 2007). ISR may activate inducible defence mechanisms in the plant in a similar way to the response against pathogenic microorganisms in incompatible interactions. These mechanisms include biochemical changes, including the reinforcements of plant cell walls, production of antimicrobial phytoalexins and synthesis of pathogenesis-related (PR) proteins, such as chitinases, β-1,3-glucanases or peroxidases (Ramamoorthy *et al*., 2001; van Loon *et al*., 2006). ISR is typically independent of salicylic acid (SA) and is mostly dependent on the jasmonate (JA) and/or ethylene (ET) signalling pathways (Verhagen *et al*., 2004; Pieterse *et al*., 2009). However, the fact that some ISR inducers also appear to activate an SA-dependent pathway indicates that different signalling pathways may operate when ISR is elicited (Ryu *et al*., 2003; Niu *et al*., 2011).

In ISR, however, the enhanced defensive capacity cannot be attributed to a direct activation of the defence-related genes. Instead, it is based on a faster and stronger activation of basal defence mechanisms when an induced plant is exposed to microbial pathogens or herbivorous insects, a phenomenon called priming (Conrath *et al*., 2002). Thus, the priming mechanism allows the plant to more effectively react to pathogens and explains the broad-spectrum action of ISR (Conrath *et al*., 2006). The phenomenon of priming is interesting for the development of new disease control methods because priming provides broad-spectrum disease resistance without significantly affecting growth and fruit or seed set (van Hulten *et al*., 2006).

In a recent report we demonstrated the ability of UMAF6639 to promote the growth of melon seedlings and provide protection against cucurbit powdery mildew by means of ISR (García-Gutiérrez *et al*., 2012). In the present study, we analysed the melon signalling pathways and defence mechanisms stimulated by UMAF6639 in response to *P. fusca*. In addition, we investigated the role of lipopeptides produced by UMAF6639 as bacterial determinants for ISR elicitation in melon plants. Our results reinforce the biotechnological potential of strain UMAF6639 both as an antagonistic agent and as an inducer of systemic resistance.

## Results

### *B. subtilis* UMAF6639 provides protection against cucurbit powdery mildew via activation of JA- and SA-dependent signalling

**Figure 1:**
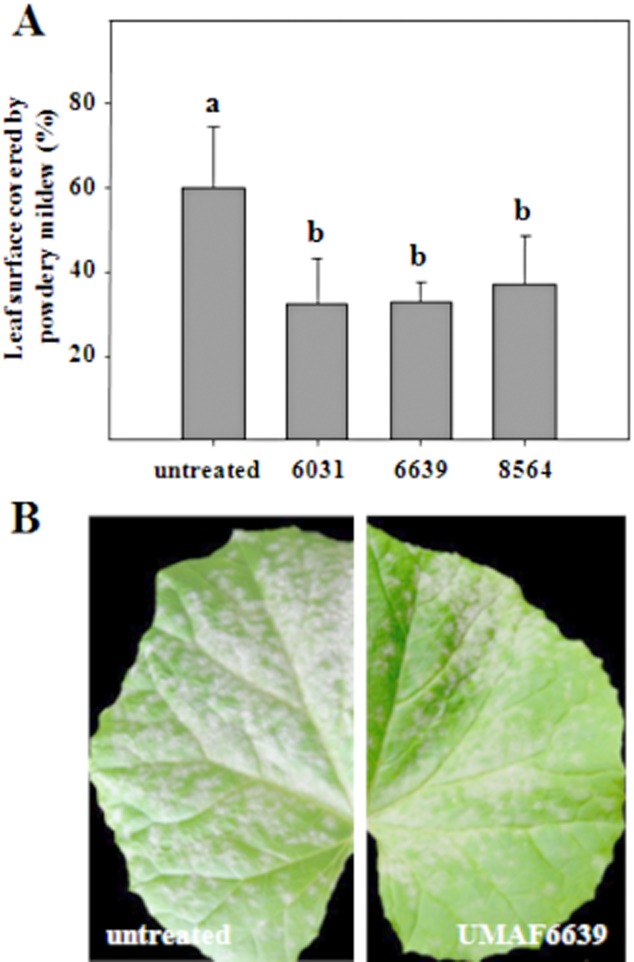
Suppressive effect of UMAF6639 towards cucurbit powdery mildew. A. Melon plants were bacterized and inoculated with *P. fusca* as described in *Experimental procedures*. Disease severity expressed as the percentage of the leaf surface covered by powdery mildew was recorded 18 days after pathogen challenge. Data represent the means of at least three independent experiments, and bars show the standard deviation. Treatments with the same letter are not significantly different at *P* = 0.05, according to Fisher's least-significant-difference test. A set of 15–20 plants was tested per treatment. B. Reduction of powdery mildew symptoms in melon seedlings by treatments with UMAF6639 following induction of a systemic resistance. Pictures were taken 18 days after inoculation with the fungal pathogen. Pictures: Untreated, leaf taken from an untreated plant, showing the upper surface completely covered by powdery mildew. UMAF6639, leaf taken from a plant treated with *B. subtilis* UMAF6639 showing significant reduction of powdery mildew symptoms.

Despite its phyllospheric origin, UMAF6639 also provides protection against powdery mildew by means of ISR (Romero *et al*., 2004; García-Gutiérrez *et al*., 2012). The application of UMAF6639 to the roots of melon seedlings provoked a significant reduction in the disease severity (about 50%) 18 days after inoculation with the fungal pathogen, which was very similar to the protection provided by the rhizospheric strains *Pseudomonas fluorescens* UMAF6031 and *Bacillus cereus* UMAF8564 (Fig. 1A). This protective effect is illustrated in Fig. 1B, which shows the reduction of symptoms in a leaf of an UMAF6639-treated plant compared with an untreated control.

**Figure 2:**
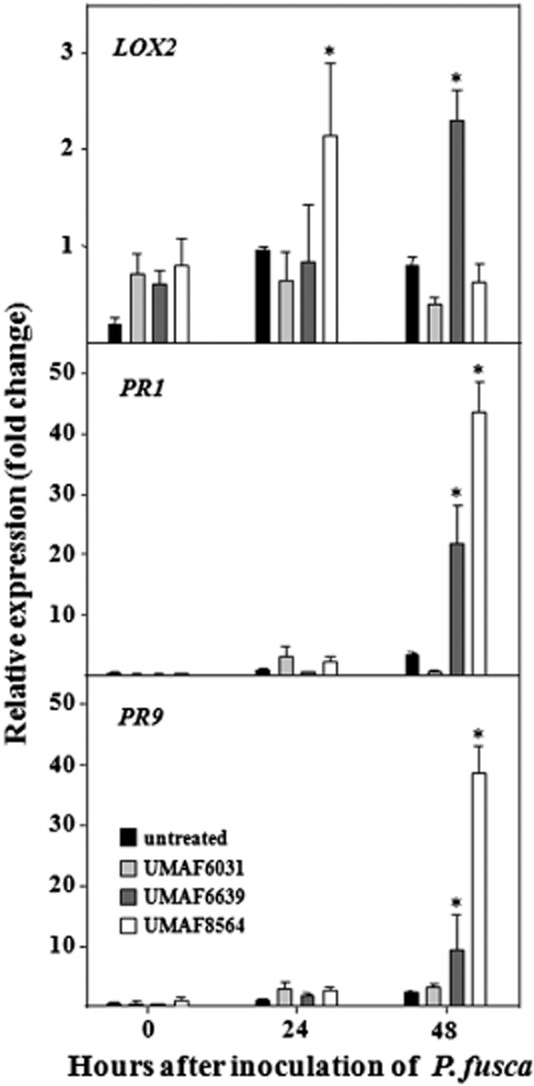
Expression of plant defence genes in bacterized melon plants in response to powdery mildew. Plants were bacterized and inoculated with *P. fusca* as described in *Experimental procedures*. Total RNA was isolated at different time points, and the relative expression of *LOX2* (lipoxygenase 2), *PR-1* and *PR-9* (peroxidase) genes was analysed by quantitative RT-PCR. Expression levels were normalized to the endogenous control gen *ACT1* (actin). Relative expression was calibrated to the untreated control 24 h post inoculation. Data shown represent average values from three independent experiments, with error bars depicting standard error. Asterisks indicate statistically significant different gene expression levels compared with untreated control (LSD test; *P* = 0.05).

To determine the signalling pathways elicited by *B. subtilis* UMAF6639 and the other two ISR-inducing strains, expression of *LOX2* (lipoxygenase 2, a JA-responsive marker gene), *PR1* (an SA-responsive marker gene) and *PR9* (peroxidase, a gene related to the hypersensitive response and cell wall reinforcement and inducible by SA and JA) (Durrant and Dong, 2004; van Loon *et al*., 2006; Pieterse *et al*., 2009) was analysed using quantitative RT-PCR (Fig. 2). In these assays, leaf samples of bacterized and non-bacterized melon plants were collected before (time 0), and 24 or 48 h after inoculation with *P. fusca*. Before inoculation with the pathogen, bacterized plants displayed a slight but not significant increase in the expression of *LOX2* compared with non-bacterized control plants (Fig. 2, upper panel). After inoculation, the expression of this gene was increased only in plants treated with *Bacillus* species. A maximum twofold increase in signal was reached 24 h and 48 h after inoculation of the pathogen in the case of *B. cereus* UMAF8564 and *B. subtilis* UMAF6639 respectively. The expression of the other two PR genes (*PR1* and *PR9*) was triggered 48 h after inoculation of *P. fusca* and also in *Bacillus*-treated plants (Fig. 2, middle and bottom panels respectively). In these cases, the highest expression level (40-fold increase) was elicited after treatment with *B. cereus* UMAF8564. The increased expression of *PR1*, which is a typical SA-responsive marker gene, suggested that the ISR response induced by the *Bacillus* strains was dependent on SA signalling. However, the limited increase of expression observed for *LOX2* did not convincingly reflect the dependence on JA signalling of such a response.

**Figure 3:**
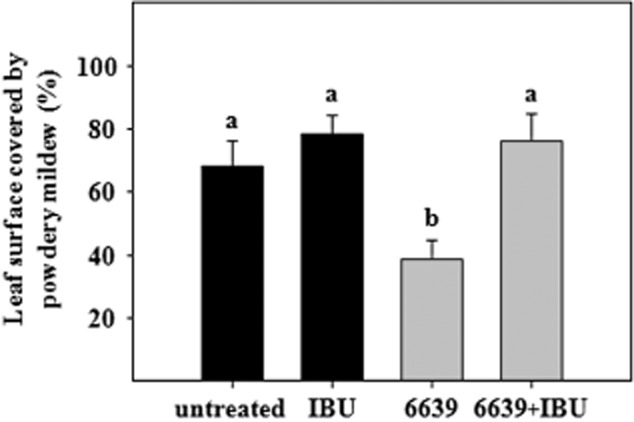
Effect of ibuprofen on systemic resistance induced by *B. subtilis* UMAF6639. ISR assays were performed essentially as described in *Experimental procedures* using melon plants bacterized with UMAF6639 and *P. fusca* as the challenging pathogen. A 5 mM solution of ibuprofen (IBU) was spread over the upper surface of the first leaf of the melon plants 24 h before inoculation of the fungal pathogen. Disease severity (the percentage of leaf surface covered by powdery mildew) was recorded 18 days after pathogen challenge. Data represent the means of three independent experiments, and bars show the standard error. Data values followed by the same letter are not significantly different at *P* = 0.05, according to Fisher's least-significant-difference test. A set of 15–20 plants was tested per treatment.

To clarify the role of JA signalling in the ISR response observed in melon by the *Bacillus* strains, the lipoxygenase inhibitor ibuprofen (IBU) was used as an antagonist of JA-dependent defence responses (Fig. 3). IBU did not affect to the development of powdery mildew symptoms in non-bacterized plants. However, disease protection was suppressed in UMAF6639-treated and IBU-exposed plants. Bacterized plants not exposed to IBU displayed disease reductions of ∼ 50%, a disease suppression that was arrested in the presence of 5 mM IBU. These results suggested that the ISR response elicited by *B. subtilis* UMAF6639 is also dependent on JA signalling.

### *B. subtilis* UMAF6639 induces production of H_2_O_2_ and cell wall strengthening in infected leaves

**Figure 4:**
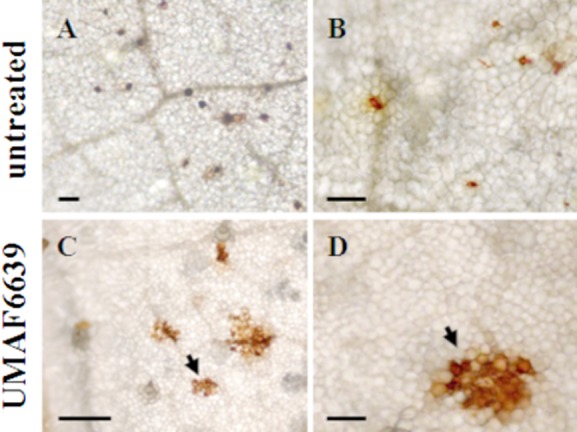
Histochemical analysis of the production reactive oxygen species in the leaves of melon plants bacterized with *B. subtilis* UMAF6639 and infected by powdery mildew. Melon plants were bacterized and inoculated with *P. fusca* as described in *Experimental procedures*. Detection of hydrogen peroxide (H_2_O_2_) was performed according to the DAB-uptake method, using bright light microscopy. Arrowheads indicate epidermal cells accumulating reddish-brown precipitates due to H_2_O_2_ production. Pictures were taken 72 h after inoculation of the fungal pathogen. Scale bars represent 50 μm (A) and 100 μm (rest of the plates).

From previous findings it appeared that *B. subtilis* UMAF6639 triggers the SA and JA pathways which may be related to the ISR response. Thus, we asked which defence mechanisms of melon plants were involved in the ISR triggered after bacterization with UMAF6639 and exposition to *P. fusca*. The production of reactive oxygen species and the accumulation of cell wall deposits were histochemically examined in leaves of melon plants previously bacterized with *B. subtilis* UMAF6639 and subsequently inoculated with *P. fusca*. Representative pictures of the production of H_2_O_2_ by epidermal cells are shown in Fig. 4. The number of cells accumulating H_2_O_2_ was higher in bacterized than in untreated controls, 72 h after the inoculation of *P. fusca*. In addition, we observed that reacting cells appeared disperse in the leaves of untreated plants, while they clustered in the leaves of the UMAF6639-treated plants. This noticeable spatial distribution of reacting cells was interpreted as a response reaction at pathogen penetration sites.

**Figure 5:**
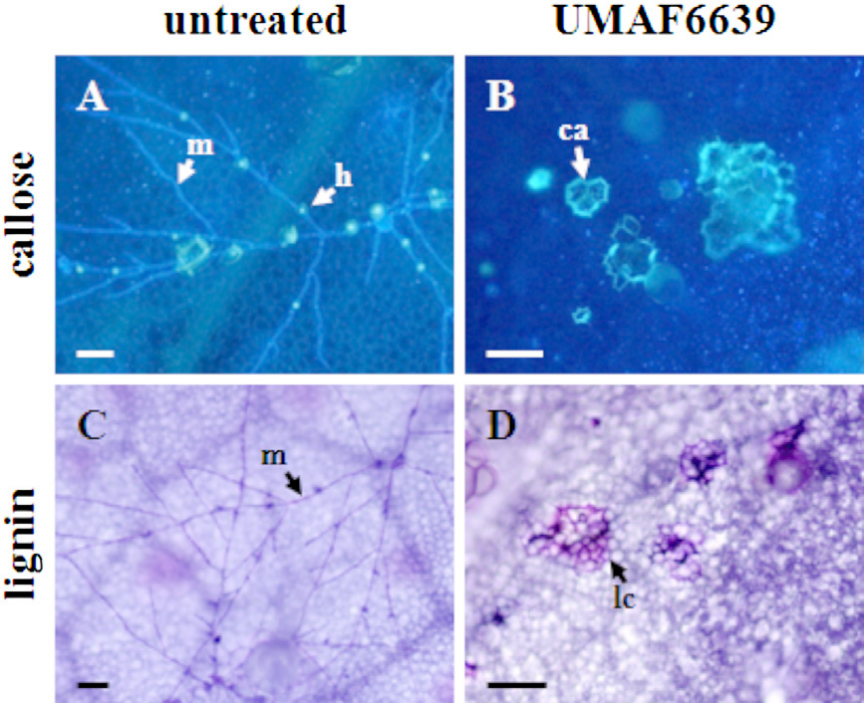
Histochemical analysis of cell wall reinforcement in leaves of melon plants bacterized with *B. subtilis* UMAF6639 and infected by powdery mildew. Melon plants were bacterized and inoculated with *P. fusca* as described in *Experimental procedures*. A and B. Detection of callose deposits (ca) surrounding the cells by calcofluor staining and fluorescence microscopy. Haustoria (h) can be distinguished as blue fluorescent spots along *P. fusca* hyphae (m). C and D. Lignin deposition analysed by toluidine staining and bright-light microscopy. Micrographs show lignified cells (lc) and *P. fusca* mycelia (m) both stained in violet. Pictures were taken 72 h after inoculation of the fungal pathogen. Scale bars represent 500 μm (B) and 50 μm (rest of the plates).

Similar results were observed when cell wall reinforcement was analysed (Fig. 5). The cell wall deposits of callose and lignin were more abundant in leaves of bacterized than non-bacterized plants 72 h after fungal inoculation. In addition, as observed for H_2_O_2_ production, these deposits were rarely found in control plants, and in plants induced with *B. subtilis* UMAF6639, callose and lignin depositions were found both in epidermal and in mesophyll cells possibly neighbouring the penetrated epidermal cells. Because no differences in cell defence responses were observed between bacterized and non-bacterized plants in the absence of *P. fusca* (data not shown), these results indicated that the activation of defence mechanisms in powdery mildew-sensitive plants in response to *P. fusca* was a consequence of a previous priming induction by *B. subtilis* UMAF6639 instead of a direct activation by the challenging pathogen.

### Surfactin is a major determinant for ISR elicitation in melon

**Figure 6:**
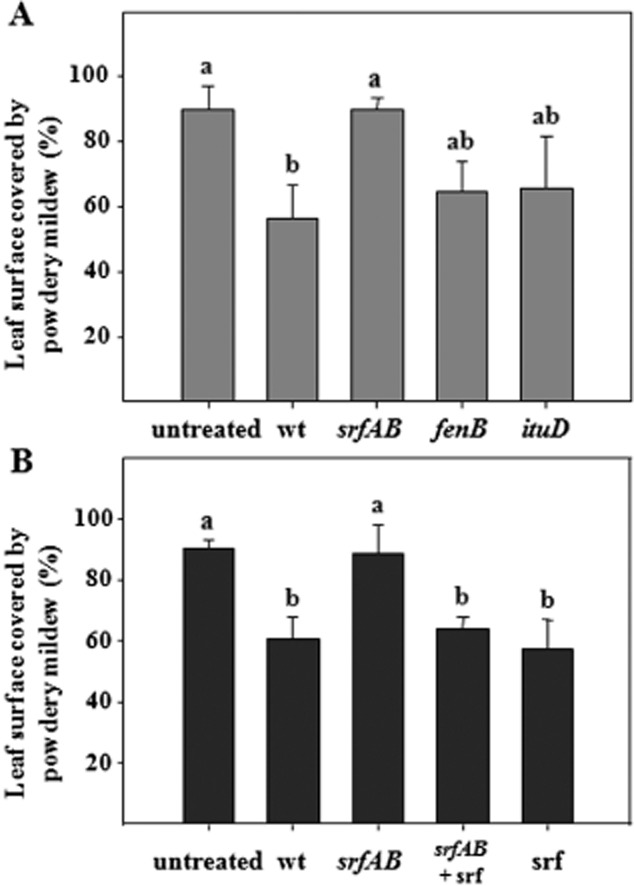
Effect of lipopeptides in the activation of ISR in melon plants against powdery mildew. A. ISR assays using mutants defective in the production of lipopeptides. Melon plants were bacterized with the wild-type strain *B. subtilis* UMAF6639 and its derivative mutants defective in the production of surfactin, fengycin or iturin A, and inoculated with *P. fusca* conidia as described in *Experimental procedures*. B. ISR assays using commercial surfactin. Melon plants were bacterized with wild-type *B. subtilis* UMAF6639, its surfactin-defective mutant or watered with 10 ml of a solution of 10 μM synthetic surfactin C_15_. Disease severity expressed as the percentage of leaf surface covered by powdery mildew was recorded 18 days after pathogen challenge. Data represent the means of at least three independent experiments, and bars show the standard deviation. Treatments with the same letter are not significantly different at *P* = 0.05, according to Fisher's least-significant-difference test. A set of 15–20 plants was tested per treatment. Abbreviations: wt, wild-type *B. subtilis* UMAF6639; *srfAB*, surfactin-deficient mutant; *fenB*, fengycin-defective derivative; *ituD*, iturin A-deficient transformant; srf, synthetic surfactin.

Lipopeptides from the fengycin and surfactin families are elicitors of ISR activation in bean and tomato plants (Ongena *et al*., 2007). According to previous studies, UMAF6639 produces lipopeptides of both families. Thus, it was tempting to speculate their involvement in the ISR trigger in melon plants. To test this hypothesis, lipopeptide-deficient derivatives of *B. subtilis* UMAF6639 were tested as inducers of ISR in melon plants against powdery mildew (Fig. 6). These single mutants are defective in the production of either fengycin, iturin A or surfactin lipopeptides (Table 1). Plants treated with mutants defective in the synthesis of iturin A (*ituD*) or fengycin (*fenB*) exhibited levels of disease protection not significantly different from those observed in plants bacterized with the wild-type strain *B. subtilis* UMAF6639. However, plants exposed to the surfactin-defective mutant (*srfAB*) displayed similar disease levels to the untreated plants (Fig. 6A). These results suggested a major role for surfactin in the ISR elicitation of melon against powdery mildew.

**Table 1:** Bacterial and fungal strains used in this study

Microorganism	Relevant characteristics	Reference
**Bacterial strains**		
*Bacillus subtilis* UMAF6639	Wild-type strain, producer of iturin A, fengycin and surfactin	Romero *et al*. (2007b)
*B. subtilis* UMAF6639::ituD	Iturin A-deficient transformant, cm^R^	Romero *et al*. (2007b)
*B. subtilis* UMAF6639::fenB	Fengycin-deficient transformant, cm^R^	Romero *et al*. (2007b)
*B. subtilis* UMAF6639::srfAB	Surfactin-deficient transformant, ery^R^	Zeriouh (2012)
*Bacillus cereus* UMAF8564	ISR inducer	García-Gutiérrez *et al*. (2012)
*Pseudomonas fluorescens* UMAF6031	ISR inducer	García-Gutiérrez *et al*. (2012)
**Phytopathogenic fungi**		
*Podosphaera fusca* SF48	Causes cucurbit powdery mildew	Romero *et al*. (2007b)

cm^R^, cloramphenicol resistant; ery^R^, erythromycin resistant.

To conclusively demonstrate the role of surfactin as a priming determinant for ISR elicitation in melon, commercial synthetic surfactin C_15_ was used in ISR assays similar to those previously performed with bacterial cells (Fig. 6B). The commercial surfactin restored the ability of the surfactin-defective mutant to reduce powdery mildew disease symptoms at levels statistically similar to the wild-type strain. Similarly, treatments with only synthetic surfactin also protected melon plants at levels observed for the wild-type strain and the combination surfactin mutant plus commercial surfactin. These results clearly demonstrated the essential role of surfactin in the activation of ISR defence mechanisms in melon plants against powdery mildew.

## Discussion

*Bacillus subtilis* UMAF6639 is a very promising biological control agent both as an antagonistic strain and as an inducer of systemic resistance in the host plant (Romero *et al*., 2007c; García-Gutiérrez *et al*., 2012). To increase the field performance and consistency of biocontrol agents, a detailed knowledge of the physiological functions underlying their biocontrol activity and environmental adaptations is necessary. The aim of this work was to gain insights into the physiological and molecular bases of the plant defence mechanisms activated by UMAF6639 in response to powdery mildew.

Rhizobacteria commonly activate an ISR response in plants in a JA/ET signalling-dependent manner. However, there are examples of SA-dependent and JA-independent defence responses (Pieterse *et al*., 1998; Park and Kloepper, 2000; Ton *et al*., 2002; Glazebrook *et al*., 2003; Ryu *et al*., 2003; Tjamos *et al*., 2005). The enhanced expression of the *LOX2* and especially *PR1* and *PR9* genes in plants treated with *B. subtilis* UMAF6639 or *B. cereus* UMAF8564 suggested that the *Bacillus*-induced ISR in melon against powdery mildew is SA- and JA-dependent. This idea is supported by two additional pieces of evidences: (i) the very strong expression of the *PR1* gene (the PR protein gene typically used as a marker of SA-dependent signalling) and (ii) the inhibition of disease protection provoked by the JA-signalling inhibitor ibuprofen. Our findings align with a growing number of reports that point towards a cooperative rather than antagonist activity of SA and JA/ET signalling pathways in plant protection provided by rhizobacteria (van Wees *et al*., 2000; Martínez *et al*., 2001; Spoel *et al*., 2003; León-Reyes *et al*., 2010; Niu *et al*., 2011).

Powdery mildews are biotrophic fungi that are usually sensitive to defence responses that are regulated by SA (Glazebrook, 2005; Pieterse *et al*., 2009). SAR inducers such as acibenzolar-*S*-methyl (ASM), benzothiadiazole (BTH), 2,6-dichloroisonicotinic acid (INA) and salicylic acid (SA), significantly reduce the severity of powdery mildews by activation of SA-dependent signalling pathways (Salmeron *et al*., 2002; Lin *et al*., 2009). However, the root endophyte fungus *Piriformospora indica* induces systemic resistance in *Arabidopsis* against powdery mildews mediated by JA (Stein *et al*., 2008). Moreover, the activation of JA signalling pathways in the *Arabidopsis cev1* mutant results in enhanced resistance to powdery mildew fungi (Ellis *et al*., 2002). Thus, the cooperative activity of several signalling pathways in plants likely leads to an efficient defence against powdery mildews.

Peroxidases have an important role in the defence mechanisms induced by beneficial soil-borne microorganisms (Bargabus *et al*., 2004; Shoresh *et al*., 2005; Buensanteai *et al*., 2009). Peroxidases are oxidative enzymes that contribute to the last step of the formation of lignin and hydrogen peroxide, two plant factors involved in disease resistance (Avdiushko *et al*., 1993; Niranjan Raj *et al*., 2012). Melon plants induced by *B. subtilis* UMAF6639 displayed an increase in the expression of peroxidase 48 h after inoculation with *P. fusca*. The corresponding increase in peroxidase activity may be essential for the manifestation of defence mechanisms, such as the production of reactive oxygen species and cell wall reinforcement. Indeed, production of hydrogen peroxide and deposits of callose and lignin were visualized in UMAF6639-induced plants 72 h after inoculation of the fungal pathogen. Such mechanisms are part of the hypersensitive response exhibited by resistant melon plants against *P. fusca* (Kuzuya *et al*., 2006; Romero *et al*., 2008). Thus, it is tempting to speculate that these mechanisms may also be involved in the powdery mildew disease reduction effect observed in UMAF6639-induced melon plants.

The results of gene expression analysis also indicated that the resistance observed in *Bacillus-*induced melon plants was based on the stronger and faster activation of defence genes upon pathogen attack. This phenomenon is called priming. Priming is associated with different types of induced resistance and provides the plant an enhanced capacity for rapid and effective activation of basal defence response once a pathogen is contacted. In priming, different beneficial microbe-associated molecular patterns (MAMPs) are recognized by the plant, which results in a mild but effective activation of the plant immune responses in systemic tissues upon perception of the pathogen (Conrath *et al*., 2002; van Wees *et al*., 2008). *Bacillus* MAMPs are poorly understood. Interestingly, the lipopeptide antibiotics surfactin and fengycin are proposed to interact with plant cells and stimulate the immune response against plant pathogens by the activation of ISR in different host plants (Ongena and Jacques, 2008).

*Bacillus subtilis* UMAF6639 produces, at least in liquid cultures, compounds from the three families of lipopeptides: fengycin, iturin A and surfactin (Romero *et al*., 2007b; Zeriouh *et al*., 2011). Thus, we investigated the role of these lipopeptides as MAMPs responsible for ISR activation in melon. A surfactin-deficient mutant displayed an altered ISR-inducing phenotype, with disease severity values comparable to untreated plants and statistically different from plants treated with the wild-type strain. This result suggested a major role of surfactin in the activation of ISR by *B. subtilis* UMAF6639. To conclusively determine the role of surfactin in ISR elicitation, synthetic surfactin C_15_ was used in similar assays (Kracht *et al*., 1999; Ongena *et al*., 2007; Jourdan *et al*., 2009; Nihorimbere *et al*., 2009; 2012). Accordingly, a single application of surfactin was sufficient to induce disease reductions similar to *B. subtilis* UMAF6639. These results conclusively demonstrated that surfactin is one of the signal molecules that trigger ISR in melon in response to powdery mildew.

Surfactin is a pore-forming molecule that is essential for cell-to-cell communication within *B. subtilis* populations (López *et al*., 2009). It is proposed that pores originating from surfactin in bacterial membranes cause potassium leakage that is sensed by the membrane-associated sensor kinase, KinC, which in turn activates the expression of genes involved in the production of the extracellular matrix and biofilm formation. Similarly, it has been postulated that in plant cells, surfactin may induce a disturbance or transient channelling in the plasma membrane, which in turn activates a cascade of molecular events leading to enhanced plant defence (Jourdan *et al*., 2009). Our results reinforce the role of surfactin as a broad spectrum MAMP with the potential to stimulate ISR in many host plants. In other words, surfactin lipopeptides appear as essential molecules both for bacterial cell–cell communication and for bacteria–plant communication. In this way, surfactins benefit the bacterial population and the fitness of the plants.

Additional bacterial determinants can act together with lipopeptides in the induction of systemic resistance. Volatile organic compounds, in particular 2,3 butanediol, are involved in induction of ISR by *Bacillus* (Ryu *et al*., 2004). UMAF6639 was able to produce volatile compounds as shown by inhibition of the *in vitro* growth of the phytopathogenic fungus *Rosellinia necatrix* (data not shown). Thus, we speculate that volatile compounds may also participate with surfactin in the activation of plant defence, but this remains to be tested.

**Figure 7:**
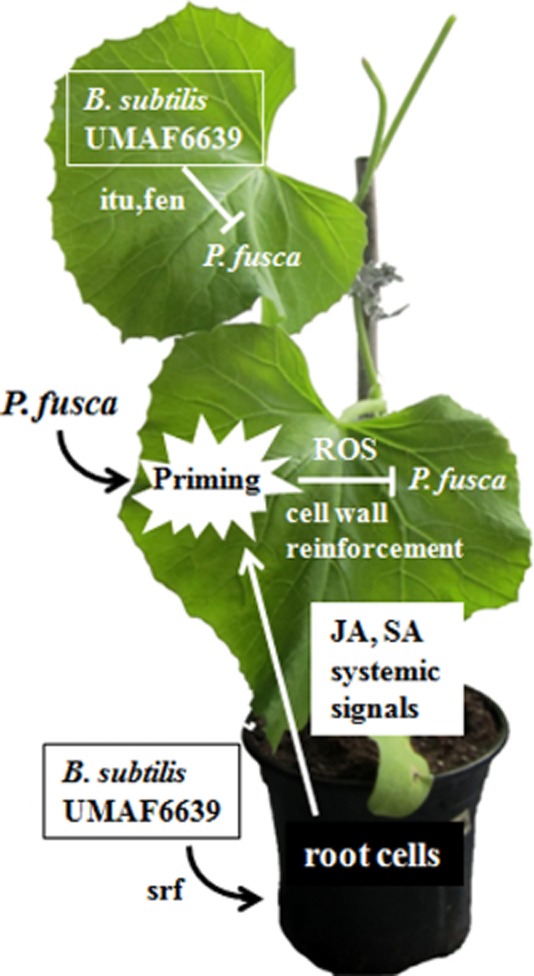
Schematic representation of the mechanisms of action of *B. subtilis* UMAF6639 against the cucurbit powdery mildew fungus *P. fusca*. UMAF6639 acts in the phyllosphere through direct antagonism mediated by the production of iturin (itu) and fengycin (fen) antifungal lipopeptides. In the rhizosphere, UMAF6639 acts by activation of JA- and SA-dependent defence responses, in which surfactin (srf) is an elicitor. These defence responses include the production of reactive oxygen species (ROS) and cell wall deposits (cell wall reinforcement), which are activated after pathogen attack (priming).

In summary, as represented in Fig. 7, *B. subtilis* UMAF6639 is able to mitigate cucurbit powdery mildew disease by at least two different mechanisms: (i) by an antagonistic action mediated by the production of the antifungal lipopeptides iturin and fengycin in the phyllosphere and (ii) by activation of JA- and SA-dependent defence responses in the rhizosphere, in which surfactin lipopeptide plays a major role as an elicitor for stimulation of the immune response. These results reinforce the biotechnological potential of *B. subtilis* UMAF6639 as both an antagonistic agent and an inducer of systemic resistance. In relation to this, sequencing of the UMAF66339 genome is currently underway. Detailed analysis of its genome should reveal a more realistic picture of the biocontrol potential of this biotechnologically interesting *Bacillus* strain.

## Experimental procedures

### Microorganisms and culture conditions

The bacterial and fungal strains used in this study are listed in Table 1. The SF48 isolate of *P. fusca* (synonym *P. xanthii*) race 1 was routinely grown on cotyledons of zucchini cv. Negro Belleza as described elsewhere (Pérez-García *et al*., 2001). *Bacillus cereus* UMAF8564, *B. subtilis* UMAF6639 and its derivative mutants were routinely grown on nutrient agar (NA) plates at 37°C. *Pseudomonas fluorescens* UMAF6031 was routinely grown on King's B medium (KB) plates at 25°C. Antibiotics, when required, were added to the culture medium at the following concentrations: 5 μg ml^−1^ chloramphenicol and 5 μg ml^−1^ erythromycin.

### ISR assays

These assays were essentially performed as previously described (García-Gutiérrez *et al*., 2012). Briefly, 2-week-old melon seedlings from cv. Rochet were used (Pérez-García *et al*., 2001). Seeds were pre-germinated in the dark, sown into pots and cultivated in a plant growth chamber until use. Before each experiment, bacterial cultures were always obtained from frozen stocks. For plant bacterization, bacterial cultures were adjusted to a cell density of 10^8^ cfu ml^−1^ without centrifugation. Plants were exposed twice to each bacterial strain. The first bacterization was performed by dipping the roots of seedlings into the corresponding bacterial suspension for 30 min. One week later, a second bacterization was performed by watering the plants with 10 ml of a cell suspension from the same bacterial strain. Inoculation of *P. fusca* was performed 1 week after the second bacterization. Conidial suspensions (10^3^ spores ml^−1^) were spread over the upper surfaces of the second and third leaves until run off. Disease symptoms were recorded 18 days after inoculation of the pathogen and are expressed as the percentage of the leaf area covered by powdery mildew (Romero *et al*., 2004).

### RNA isolation and gene expression analysis

For RNA isolation, melon leaf samples were finely ground in liquid nitrogen, and total RNA was isolated according to the TRI Reagent® RNA isolation system (Sigma-Aldrich, USA), following the manufacturer's recommendation with a minor modification. For RNA precipitation, 10 M LiCl was used instead of isopropanol. RNA concentration was determined using a NanoDrop spectrophotometer ND-1000® (Thermo Scientific, USA). Contaminating DNA was removed using a TURBO DNA-free kit® (Ambion, USA).

Quantitative RT-PCR was used to analyse gene expression. Expression of three plant defence-related genes was studied: *LOX2* (lipoxygenase 2), *PR1* (PR-1) and *PR9* (peroxidase). Expression levels were normalized to the endogenous control gen *ACT1* (actin). Primers and TaqMan probes used in this study are listed in Table 2. The qPCR reactions were assembled in a 25 μl volume containing 12.5 μl of 2× TaqMan Fast Universal Master Mix No AmpErase UNG (Applied Biosystems, USA), 0.25 μl of MultiScribe Reverse Transcriptase (Applied Biosystems), 0.5 μl of RNase Inhibitor (Applied Biosystems), 0.75 μl of 10 μM TaqMan probe (Applied Biosystems), 2 μl (10 pmol) of sense and antisense primers and 5 μl of total RNA (0.01 μg μl^−1^).

**Table 2:** Primers and TaqMan probes used in this study

Gene	Accession number	Primer or probe designation	Sequence (5′ → 3′)
*ACT1* (actin)	FJ763186	ActF	TGTCTGCAATACCAGGGAACAT
		ActR	TGTGACGTAGATATCAGAAAGGACCTT
		ActTP	ACCACCACTGAGGACGATGTTTCCGT
*LOX2* (lipoxygenase)	GQ386815	LoxF	GCGTAAGGAATGGGATAGAATATATGA
		LoxR	CGACGAGGATAAGGGAATTGG
		LoxTP	TATCTATAACGACCTTTCCGAACCCGGTGA
*PR1*	EU556704	Pr1F	GAGTGGGACAGAATAGTAGCAGGTT
		Pr1R	GTGCACTAGCCTACAGTCGTTGA
		Pr1TQ	TGCTCAACAATACGCGAACCAACGC
*PR9* (peroxidase)	AY373372	Pr9F	GCATCTCGATCGTCCAAATGT
		Pr9R	TTGGGCTCAATACCGTGGAT
		Pr9TQ	TGCGCCCCAGATAAAGCGACGA

In the names of the primers or probes F, R and TP stand for forward (F) or reverse (R) primers and TaqMan (TP) probes respectively.

### Histochemical detection of reactive oxygen species

The *in situ* accumulation of hydrogen peroxide (H_2_O_2_) was determined by histochemical analysis. Detection of H_2_O_2_ was performed according to the 3,3′-diaminobenzidine (DAB)-uptake method. Leaf disks were incubated in 0.1% DAB (pH 3.8) overnight in the dark and at room temperature. After incubation, the disks were immersed in boiling ethanol to stop the reaction and bleach the disks. Finally, the leaf disks were analysed under a light microscope for brownish-red precipitates corresponding to H_2_O_2_ accumulation (Thordal-Christensen *et al*., 1997; Romero *et al*., 2008).

### Histochemical analysis of plant cell wall strengthening

Plant cell wall reinforcement was also histochemically analysed. For these analyses, leaf samples were cleared in boiling ethanol and examined for the presence of cell wall deposits. Detection of callose-like materials was performed using a UV epifluorescence microscope according to the calcofluor staining technique. Leaf disks were stained by immersion for 24 h in a solution of 0.01% aniline blue in 7 mM K_2_HPO_4_ (pH 8.9). the disks were mounted on glass slides with 0.1% calcofluor and examined under a fluorescence microscope. Callose-like deposits appeared as fluorescent yellow-stained layers surrounding the cells, *P. fusca* structures fluoresced in blue and the haustorial penetration sites appeared as fluorescent yellow spots (Cohen *et al*., 1990; Romero *et al*., 2008). Detection of lignin deposits was performed by staining the leaf disks in 0.05% toluidine blue for 10 min. After rinsing in water, the disks were mounted on glass slides and examined under a light microscope for the occurrence of lignified cells, which were identified by violet stained staining (O'Brian *et al*., 1964; Romero *et al*., 2008).

### Statistical analysis

Data were analysed using statistics software SPSS 8.0 (SPSS, Chicago, USA). One-way analysis of variance (anova) was applied, and treatment means were separated by Fisher's least-significant-difference test (*P* = 0.05).

## Conflict of interest

Authors declare that they have no conflict of interest in relation to this work.

## References

[b1] Agrios GN (2005). Plant Pathology.

[b2] Avdiushko SA, Ye XS, Kuc J (1993). Detection of several enzymatic activities in leaf prints cucumber plant. Physiol Mol Plant Pathol.

[b3] Bargabus RL, Zidack NK, Sherwood JW, Jacobsen BJ (2004). Screening for the identification of potential biological control agents that induce systemic acquired resistance in sugar beet. Biol Control.

[b4] Bélanger RR, Labbé C, Bélanger RR, Bushnell WR, Dik AJ, Carver TLW (2002). Control of powdery mildews without chemicals: prophylactic and biological alternatives for horticultural crops. The Powdery Mildews. A Comprehensive Treatise.

[b5] Buensanteai N, Yuen GY, Prathuangwong S (2009). Priming, signalling and protein production associated with induced resistance by *Bacillus amyloliquefaciens* KPS46. World J Microbiol Biotechnol.

[b6] Cohen Y, Eyal H, Hanania J (1990). Ultraestructure, autofluorescence, callose deposition and lignifications in compatible and resistant muskmelon leaves infected with the powdery mildew fungus *Sphaerotheca fuliginea*. Physiol Mol Plant Pathol.

[b8] Conrath U, Pieterse CMJ, Mauch-Many B (2002). Priming in plant–pathogen interactions. Trends Plant Sci.

[b7] Conrath U, Beckers GJM, Flors V, García-Agustin P, Jakab G, Mauch F (2006). Priming: getting ready for battle. Mol Plant Microbe Interact.

[b9] Durrant WE, Dong X (2004). Systemic acquired resistance. Annu Rev Phytopathol.

[b10] Ellis C, Karafyllidis L, Turner JG (2002). Constitutive activation of jasmonate signalling in *Arabidopsis* mutant correlates with enhanced resistance to *Eryshiphe cichoracearum, Pseudomonas syringae* and *Myzus persicae*. Mol Plant Microbe Interact.

[b11] Francis I, Holsters M, Vereecke D (2010). The gram-positive side of plant microbe interactions. Environ Microbiol.

[b12] García-Gutiérrez L, Romero D, Zeriouh H, Cazorla FM, Torés JA, de Vicente A, Pérez-García A (2012). Isolation and selection of plant growth-promoting rhizobacteria as inducers of systemic resistance in melon. Plant Soil.

[b13] Glazebrook J (2005). Contrasting mechanisms of defence against biotrophic and necrotrophic pathogens. Annu Rev Phytopathol.

[b14] Glazebrook J, Chen W, Estes B, Chang HS, Nawrath C, Métraux JP (2003). Topology of the network integrating salycilate and jasmonate signal transduction derived from global expression phenotyping. Plant J.

[b15] Hewitt HG (1998). Fungicides in Crop Protection.

[b16] Hollomon DW, Wheeler IE, Bélanger RR, Bushnell WR, Dik AJ, Carver TLW (2002). Controlling powdery mildews with chemistry. The Powdery Mildews. A Comprehensive Treatise.

[b54] van Hulten M, Pelser M, van Loon LC, Pieterse CMJ, Ton J (2006). Costs and benefits of priming for defense in *Arabidopsis*. Proc Natl Acad Sci USA.

[b17] Jourdan E, Henry G, Dommes J, Barthélemy JP, Thonart P, Ongena M (2009). Insights into the defense-related events occurring in plant cells following perception of surfactin-type lipopeptide from *Bacillus subtilis*. Mol Plant Microbe Interact.

[b18] Kracht M, Rokos H, Ozel M, Kowall M, Pauli G, Vater J (1999). Antiviral and hemolitic activities of surfactin isoforms and their methyl ester derivatives. J Antibiot.

[b19] Kuzuya M, Yashiro K, Tomita K, Ezura H (2006). Powdery mildew (*Podosphaera xanthii*) resistance in melon is categorized into two types based on inhibition of the infection processes. J Exp Bot.

[b20] León-Reyes A, Van der Does D, de Lange ES, Delker C, Wasternack C, Van Wees SCM (2010). Salicylate-mediated suppression of jasmonate-responsive gene expression in *Arabidopsis* is targeted downstream of the jasmonate biosynthesis pathway. Planta.

[b21] Lin T-C, Ishizaka M, Ishii H (2009). Acibenzolar-S-methyl induced systemic resistance against anthracnose and powdery mildew diseases on cucumber plants without accumulation of phytoalexins. J Phytol.

[b55] van Loon LC, Bakker PAH, Pieterse CMJ (1998). Systemic resistance induced by rhizosphere bacteria. Annu Rev Phytopathol.

[b56] van Loon LC, Rep M, Pieterse CMJ (2006). Significance of inducible defense-related proteins in infected plants. Annu Rev Phytopathol.

[b22] López D, Vlamakis H, Losick R, Kolter R (2009). Cannibalism enhances biofilm development in *Bacillus subtilis*. Mol Microbiol.

[b23] Martínez C, Blanc F, le Claire E, Besnard O, Nicole M, Baccou J-C (2001). Salicylic acid and ethylene pathways are differentially activated in melon cotyledons by active or heat-denatured cellulose from *Trichoderma longibrachiatum*. Plant Physiol.

[b25] Nihorimbere V, Fickers P, Thonart P, Ongena M (2009). Ecological fitness of *Bacillus subtilis* BGS3 regarding production of the surfactin lipopeptide in the rhizosphere. Environ Microbiol Rep.

[b24] Nihorimbere V, Cawoy H, Seyer A, Brunelle A, Thonart P, Ongena M (2012). Impact of rhizosphere factors on cyclic lipopeptide signature from the plant beneficial strain *Bacillus amyloliquefaciens* S499. FEMS Microbiol Ecol.

[b26] Niranjan Raj S, Lavanya SN, Amruthesh KN, Niranjana SR, Reddy MS, Shetty HS (2012). Histo-chemical changes induced by PGPR during induction of resistance in pearl millet against downy mildew disease. Biol Control.

[b27] Niu D-D, Liu H-X, Jiang C-H, Wang Y, Wang Q-Y, Jin H-L, Guo J-H (2011). The plant growth-promoting rhizobacteria *Bacillus cereus* AR156 induces systemic resistance in *Arabidopsis thaliana* by simultaneously activating salicylate and jasmonate/ethylene-dependent signalling pathways. Mol Plant Microbe Interact.

[b28] O'Brian TP, Feder N, McCullly M (1964). Polychromatic staining of plant cell walls by toulidine blue. Protoplasma.

[b29] Ongena M, Jacques P (2008). *Bacillus* lipopeptides: versatile weapons for plant disease biocontrol. Trends Microbiol.

[b30] Ongena M, Jourdan E, Adam A, Paquot M, Brans A, Joris B (2007). Surfactin and fengycin lipopeptides of *Bacillus subtilis* as elicitors of induced systemic resistance in plants. Environ Microbiol.

[b31] Park KS, Kloepper JW (2000). Activation of *PR**1*a promoter by rhizobacteria that induce systemic resistance in tobacco against *Pseudomonas syringae* pv. *tabaci*. Biol Control.

[b32] Pérez-García A, Olalla L, Rivera ME, del Pino D, Cánovas F, de Vicente A, Torés JA (2001). Development of *Sphaerotheca fusca* on susceptible, resistant and temperature-sensitive resistant melon cultivars. Mycol Res.

[b34] Pérez-García A, Romero D, Fernández-Ortuño D, López-Ruiz FJ, de Vicente A, Torés JA (2009). The powdery mildew fungus *Podosphaera fusca* (synonym *Podosphaera xanthii*), a constant threat to cucurbits. Mol Plant Pathol.

[b33] Pérez-García A, Romero D, de Vicente A (2011). Plant protection and growth stimulation by microorganisms: biotechnological applications of Bacilli in agriculture. Curr Opin Biotechnol.

[b36] Pieterse CMJ, van Wees SCM, van Pelt JA, Knoester M, Laan R, Gerrits H (1998). A novel signalling pathway controlling induced systemic resistance in *Arabidopsis*. Plant Cell.

[b35] Pieterse CMJ, León-Reyes A, van der Ent S, Wees SCM (2009). Networking by small-molecule hormones in plant immunity. Nat Chem Biol.

[b37] Pozo MJ, Azcón-Aguilar C (2007). Unravelling mycorrhiza-induced resistance. Curr Opin Plant Biol.

[b38] Ramamoorthy V, Viswanathan R, Raguchander T, Prakasam V, Samiyappan R (2001). Induction of systemic resistance by plant growth promoting rhizobacteria in crop plants against pests and diseases. Crop Protect.

[b42] Romero D, Pérez-García A, Rivera ME, Cazorla FM, de Vicente A (2004). Isolation and evaluation of antagonist bacteria towards the cucurbit powdery mildew fungus *Podosphaera fusca*. Appl Microbiol Biotechnol.

[b39] Romero D, de Vicente A, Olmos JL, Dávila JC, Pérez-García A (2007a). Effect of lipopeptides of antagonistic strains of *Bacillus subtilis* on the morphology and ultrastructure of the cucurbit fungal pathogen *Podosphaera fusca*. J Appl Microbiol.

[b40] Romero D, de Vicente A, Rakotoal RH, Dufour SE, Veening J-W, Arrebola E (2007b). The iturin and fengycin families of lipopeptides are key factors in antagonism of *Bacillus subtilis* toward *Podosphaera fusca*. Mol Plant Microbe Interact.

[b41] Romero D, de Vicente A, Zeriouh H, Cazorla FM, Fernández-Ortuño D, Torés JA, Pérez-García A (2007c). Evaluation of biological control agents for managing cucurbit powdery mildew on greenhouse-grown melon. Plant Pathol.

[b43] Romero D, Rivera ME, Cazorla FM, Codina JC, Fernández-Ortuño D, Torés JA (2008). Comparative histochemical analyses of oxidative burst and cell wall reinforcement in compatible and incompatible melon-powdery mildew (*Podosphaera fusca*) interactions. J Plant Physiol.

[b45] Ryu C-M, Hu CH, Reddy MS, Kloepper JW (2003). Different signalling pathways of induced resistance by rhizobacteria in *Arabidopsis thaliana* against two pathovars of *Pseudomonas syringae*. New Phytol.

[b44] Ryu C-M, Farag MA, Hu CH, Reddy MS, Kloepper JW, Paré PW (2004). Bacterial volatiles induce systemic resistance in *Arabidopsis*. Plant Physiol.

[b46] Salmeron J, Vernooij B, Lawton K, Kramer C, Frye C, Osterndorp M, Bélanger RR, Bushnell WR, Dik AJ, Carver TLW (2002). Powdery mildew control through transgenic expression of antifungal proteins, resistance genes and systemic acquired resistance. The Powdery Mildews. A Comprehensive Treatise.

[b47] Schallmey M, Singh A, Ward OP (2004). Developments in the use of *Bacillus* species for industrial production. Can J Microbiol.

[b48] Shoresh M, Yedidia I, Chet I (2005). Involvement of jasmonic acid/ethylene signalling pathway in the systemic resistance induced in cucumber by *Trichoderma asperellum* T203. Phytopathology.

[b49] Spoel SH, Koornneef A, Claessens SMC, Korzelius JP, van Pelt JA, Mueller MJ (2003). NPR1 modulates cross-talk between salicylate- and jasmonate-dependent defense pathways through a novel function in the cytosol. Plant Cell.

[b50] Stein E, Molitor A, Kogel KH, Waller F (2008). Systemic resistance conferred by the mycorrhizal fungus *Piriformospora indica* requires jasmonic acid signalling and the cytoplasmic function of NPR1. Plant Cell Physiol.

[b51] Thordal-Christensen H, Zhang Z, Wei Y, Collinge DB (1997). Subcellular localization of H_2_O_2_ accumulation in papillae and hypersensitive response during the barley-powdery mildew interaction. Plant J.

[b52] Tjamos SE, Flemetakis E, Paplomatas EJ, Katinakis P (2005). Induction of resistance of *Verticillium dahliae* in *Arabidopsis thaliana* by the biocontrol agent K-165 and pathogenesis-related proteins gene expression. Mol Plant Microbe Interact.

[b53] Ton J, van Pelt JA, van Loon LC, Pieterse CMJ (2002). Differential effectiveness of salicylate-dependent and jasmonate/ethylene-dependent induced resistance in *Arabidopsis*. Mol Plant Microbe Interact.

[b59] Verhagen BWM, Glazebrook J, Zhu T, Chang HS, van Loon LC, Pieterse CMJ (2004). The transcriptome of rhizobacteria-induced systemic resistance in *Arabidopsis*. Mol Plant Microbe Interact.

[b57] van Wees SCM, de Swart EAM, van Pelt JA, van Loon LC, Pieterse CMJ (2000). Enhanced of induced disease resistance by simultaneous activation of salicylate and jasmonate-dependent defense pathways in *Arabidopsis thaliana*. Proc Natl Acad Sci USA.

[b58] van Wees SCM, van der Ent S, Pieterse CMJ (2008). Plant immune responses triggered by beneficial microbes. Curr Opin Plant Biol.

[b60] Zeriouh H (2012). Mecanismos de acción y determinantes bacterianos implicados en la actividad de biocontrol de Bacillus.

[b61] Zeriouh H, Romero D, García-Gutiérrez L, Cazorla FM, de Vicente A, Pérez-García A (2011). The iturin-like lipopeptides are essential components in the biological control arsenal of *Bacillus subtilis* against bacterial diseases of cucurbits. Mol Plant Microbe Interact.

